# Pharmacological inhibition of nSMase2 reduces brain exosome release and α-synuclein pathology in a Parkinson’s disease model

**DOI:** 10.1186/s13041-021-00776-9

**Published:** 2021-04-19

**Authors:** Chunni Zhu, Tina Bilousova, Samantha Focht, Michael Jun, Chris Jean Elias, Mikhail Melnik, Sujyoti Chandra, Jesus Campagna, Whitaker Cohn, Asa Hatami, Patricia Spilman, Karen Hoppens Gylys, Varghese John

**Affiliations:** 1 Drug Discovery Lab, Department of Neurology, University of California, Los Angeles, CA 90095 USA; 2 School of Nursing, University of California, Los Angeles, CA 90095 USA

**Keywords:** Parkinson’s disease, Alpha-synuclein, Extracellular vesicles, Exosomes, Neutral sphingomyelinase-2

## Abstract

**Aim:**

We have previously reported that cambinol (DDL-112), a known inhibitor of neutral sphingomyelinase-2 (nSMase2), suppressed extracellular vesicle (EV)/exosome production in vitro in a cell model and reduced tau seed propagation. The enzyme nSMase2 is involved in the production of exosomes carrying proteopathic seeds and could contribute to cell-to-cell transmission of pathological protein aggregates implicated in neurodegenerative diseases such as Parkinson’s disease (PD). Here, we performed in vivo studies to determine if DDL-112 can reduce brain EV/exosome production and proteopathic alpha synuclein (αSyn) spread in a PD mouse model.

**Methods:**

The acute effects of single-dose treatment with DDL-112 on interleukin-1β-induced extracellular vesicle (EV) release in brain tissue of Thy1-αSyn PD model mice and chronic effects of 5 week DDL-112 treatment on behavioral/motor function and proteinase K-resistant αSyn aggregates in the PD model were determined.

**Results/discussion:**

In the acute study, pre-treatment with DDL-112 reduced EV/exosome biogenesis and in the chronic study, treatment with DDL-112 was associated with a reduction in αSyn aggregates in the substantia nigra and improvement in motor function. Inhibition of nSMase2 thus offers a new approach to therapeutic development for neurodegenerative diseases with the potential to reduce the spread of disease-specific proteopathic proteins.

**Supplementary Information:**

The online version contains supplementary material available at 10.1186/s13041-021-00776-9.

Neurodegenerative diseases such as tauopathies and synucleinopathies [1–3] are typically characterized by the spread of proteopathic aggregates throughout the brain [4, 5]. Pathological protein aggregates comprising tau in tauopathies or alpha-synuclein (αSyn) in Parkinson’s disease (PD) [6] first appear in a specific brain region and, as disease progresses, spread to other areas of the brain following neuroanatomical pathways.

Exosomes, small (30–150 nm in diameter) extracellular vesicles (EVs) of endocytic origin [7, 8], have been implicated in the spread of protein aggregates throughout the brain [9] and specifically in the the propagation of αSyn pathology [10–16]. A subset of exosomes generated by a pathway independent of the major canonical endosomal sorting complexes required for transport (ESCRT) [17], is dependent on the activity of neutral sphingomyelinase 2 (nSMase2) and plays a key role in the spread of proteopathic seeds [18]. nSMase2 inhibition has been shown to also be associated with reduction in amyloid plaque load and tau pathology in murine models of Alzheimer’s disease [19] and tauopathy [20], respectively.

In Bilousova et al*.* 2018, we reported on identification of a small molecule inhibitor of nSMase2, cambinol (DDL-112), that decreased tau propagation, through screening of a small compound library in a cell model [21]. We showed that DDL-112, inhibits nSMase2 activity with an IC50 ~ 7.7 μM in vitro and also inhibits the nSMase2 activity in the brain after a single oral dose [22]. nSMase2 hydrolyzes sphingomyelin to produce ceramide and thereby contributes to EV/exosome formation in the brain [23]. The role of nSMase2 in the development or progression of neurodegenerative disorders is evidenced by increased ceramide levels in the brain, serum and/or plasma that have been reported as early predictors of such disorders [24] and memory impairment in PD [25].

Here, we extend our in vitro findings by assessment of the in vivo effects of DDL-112 on EV biogenesis in an acute study, and on behavior/motor function and αSyn agregates in the Thy1-αSyn PD mouse model [26–28] in a chronic study.

In the studies described below, sucrose gradient purification was used for EV/exosome isolation from brain tissue based on published protocols [29–33] with minor modifications. Characterization of sucrose gradient F1, F2, and F3 fractions is presented in Additional file 1: Fig. S1. Immunoblot analysis confirmed the prevalence of exosomal markers CD63 and syntenin-1 (Synt-1), but no negative control marker calnexin (CNX), in the F2 fraction as compared to the F1 and F3 fractions (Additional file 1: Fig. S1A). Transmission electron microscopy (TEM) of F1, F2, and F3 fractions from mouse brain EV/exosome purification shows an abundance of small EVs with the characteristic ‘cup’ shape and size of ~ 30–150 nm in the F2 fraction (Fig. 1b and Additional file 1: Fig. S1B). Small EVs (< 50 nm) were also found in the F3 fraction, but at a very low concentration and the F1 fraction largely consisted of membranous debris (Additional file 1: Fig. S1B).

**Fig. 1:**
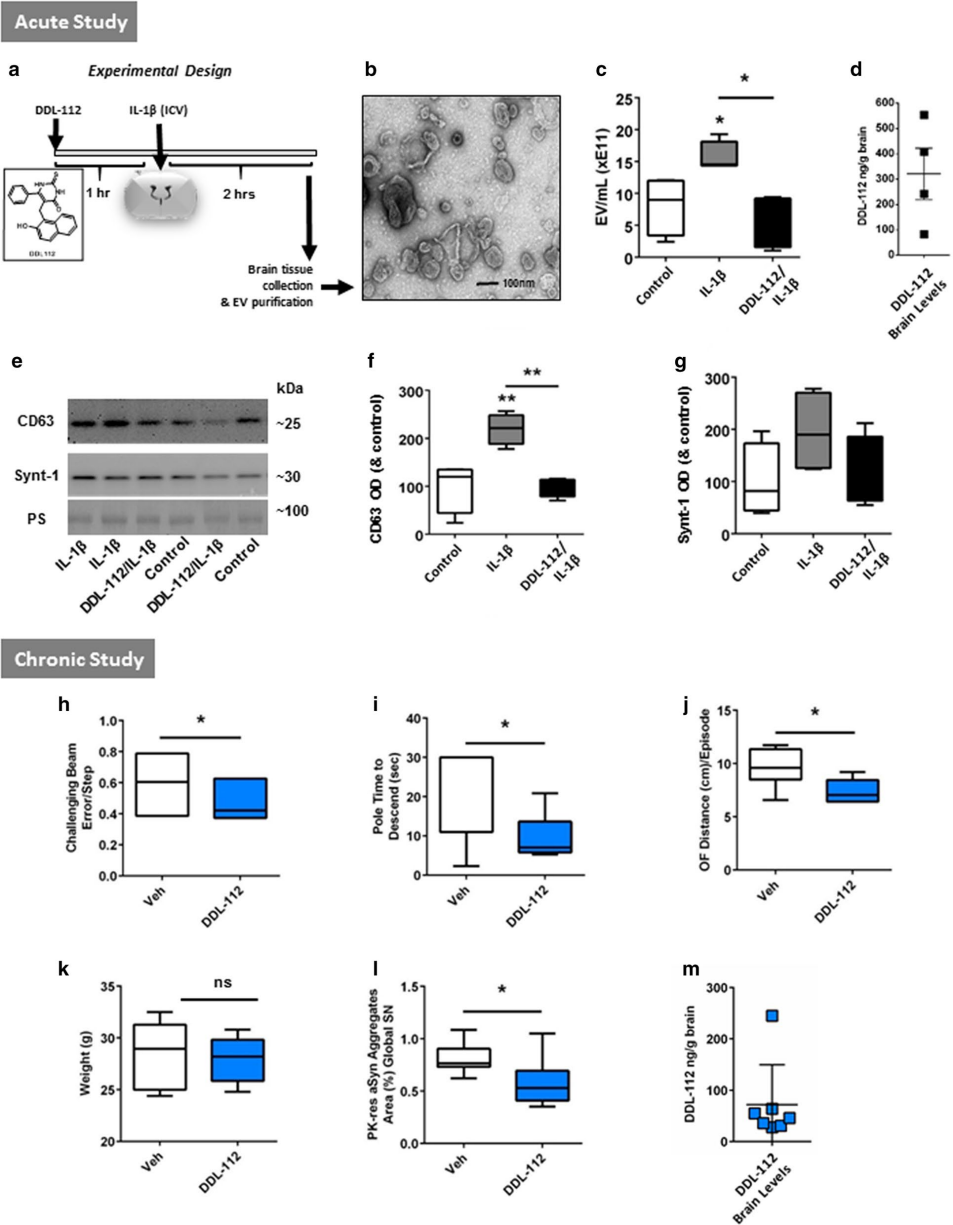
Inhibition of EV release by DDL-112 in an acute study and improvement in motor function as well as reduction of PK-resistant αSyn aggregates in the SN with chronic DDL-112 treatment of PD model mice. Acute study: **a** A scheme of the acute study protocol wherein mice were pre-treated with DDL-112 then received ICV injection of IL-1β before tissue collection is shown. **b** A representative transmission electron microscopy (TEM) image of the brain EV fraction. **c** Average concentrations of 50–200 nm size EVs from each treatment condition compared by Tunable Resistive Pulse Sensing (TRPS) analysis and levels. **d** Levels of DDL-112 in brain tissue of mice represented by the mean and SEM. **e** Representative images of immunoblot (IB) analysis of EV fractions from individual animals are shown; membranes were probed against exosomal markers (CD63 and Synt-1) with Ponceau S (PS) as the loading control. **f** Densitometry analysis of CD63 IB images. **g** Densitometry analysis of Syntenin-1 (Synt-1) IB images. Optical density (OD) is shown as percent of control. N = 4 animals per group. Statistical analysis was performed using one-way ANOVA with post-hoc Tukey comparison tests: *p < 0.05 and **p < 0.01. Chronic study: Behavioral/motor function assessment of **h** Challenging Beam (CB) error-step (Veh n = 8, DDL-112 n = 7), **i** pole test time to descend (Veh n = 7, DDL-112 n = 6; one mouse in each group was a non-performer), and **j** Open Field (OF) distance (cm) traveled per episode of movement (Veh n = 8, DDL-112 n = 6; on mouse in the DDL-112 group was a non-performer). **k** Animal weight with and without DDL-112 treatment is shown. **l** The area in percent with proteinase K (PK)-resistant αSyn aggregates in the global SN is graphed. (Veh n = 8, DDL-112 n = 7). **m** Brain levels of DDL-112 at the time of euthanasia (4 h after dosing) are shown. All data plotted minimum to maximum. Statistics performed using Student's unpaired t-test (*p < 0.05; **p < 0.01; ***p < 0.001; ****p < 0.0001)

The sucrose gradient characterization data confirm the enrichment of EV/exosomes in the F2 sucrose gradient fractions. To normalize these EV-containing F2 fractions for analyses, F2 pellets were resuspended in volumes of cryopreservation solution based on the original brain tissue weight (0.4 g of tissue/150 µl solution). Further details of the in vivo experimental methods done under protocols approved by the Animal Care and Use Committee can be found in Additional file 1.

To inform design of both the acute and chronic studies, we performed a preliminary study using Thy1-αSyn (Tg) and non-transgenic (NTg) littermate mice wherein mice were dosed orally with 100 mg/kg DDL-112 and brain tissue collected 3 h later for the determination of DDL-112 brain levels and analysis of EV/exosomes. The mean DDL-112 brain tissue level for both Tg and NTg mice was ~ 650 ng/g (Additional file 1: Fig. S1C). The means were lower for EV/exosome levels in brain tissue from DDL-112-treated Tg (but not NTg) compared to DMSO vehicle-treated mice. Furthermore, EV αSyn (pS129) levels from DDL-112-treated Tg mice were lower when compared to vehicle, although the differences were not statistically significant (Additional file 1: Fig. S1D). Levels of exosomal marker CD63 in F2 fractions from DDL-112-treated Tg mice were significantly lower compared to vehicle (Additional file 1: Fig. S1E, F).

The findings from the preliminary study (Additional file 1: Fig. S1) suggest that EV/exosome biogenesis in Thy1-αSyn Tg mice is nSMase2-dependent, but also revealed that evaluation of nSMase2 inhibitors in an acute setting would benefit from additional nSMase2 stimulation in Thy1-αSyn mice. Thus, in the acute study, after oral dosing pretreatment with DDL-112, mice were injected intracerebroventricularly (ICV) with interleukin-1β (IL-1β) known to activate nSMase2 [34, 35] and elicit release of EVs [36, 37]. We chose this method of eliciting EV release because of the reported effect of IL-1β to activate nSMase2, polymorphisms in IL-1β are associated with increased risk for PD [38], and neuroinflammation mediated by IL-1β increases suseptability of dopaminergic neurons to degeneration in animal models [39]; thus IL-1β could induce an PD phenotype [40].

In the chronic study, Thy1-αSyn mice were treated orally with DDL-112 for 5 weeks but without any IL-1β stimulation as the goal of this study was to assess the long-term (5 week) effects of nSmase2 inhibition on behavior/motor function and proteinase K-resistant αSyn aggregate load in a key area of brain affected by αSyn, the *substantia nigra* (SN).

As indicated in Fig. 1a showing the experimental design of the acute study, male Thy1-αSyn PD model mice [26] received a single oral gavage dose of DDL-112 at 100 mg/kg, then one hour later were deeply anesthetized to receive 2 ng IL-1β ICV; two hours later, this group (DDL-112) mice were euthanized and brain tissue collected for the isolation of EVs for analysis. Other groups in the study included vehicle-only ICV injection (Control) and IL-1β ICV injection without DDL-112 pre-treatment (IL-1β). Further details of the experimental methods used in the acute in vivo study, performed using protocols approved by the Animal Care and Use Committee, can be found in Additional file 1.

EVs of 50–200 nm in size from each treatment condition were compared by Tunable Resistive Pulse Sensing (TRPS) analysis, which revealed that IL-1β ICV injection significantly increased EV release (p < 0.05) and that DDL-112 pre-treatment significantly (p < 0.05) suppressed the IL-1β-induced increase in EV/exosomes as shown in Fig. 1c. In the acute study, DDL-112 inhibition of IL-1β-induced EV/exosome release was seen in the presence of mean brain level of ~ 320 ng/g measured 3 h after dosing (Fig. 1d).

The EV/exosomes from the acute study mice were also analyzed by immunoblot, using probes for Synt-1 and pan-exosomal marker CD63. Representative blots are shown in Fig. 1e and Additional file 1: Figure S2A–C. CD63 was significantly increased in EV fractions by IL-1β injection (p < 0.01), and this increase was significantly decreased (p < 0.01) by DDL-112 pretreatment (Fig. 1f). Levels of Synt-1, a marker more specific for a subpopulation of exosomes generated through the syndecan-syntenin pathway [41], while not significantly different between groups, showed a similar pattern of mean levels being higher in the IL-1β treated group as compared to DDL-112 treated but with greater variability amongst mice (Fig. 1g).

In the collected EV/exosomes, while the αSyn levels were lower we did not see a significant difference among the fractions. A representative immunoblot for EV fractions probed with anti-human αSyn and densitometry analysis, and Ponceau S staining of the membrane are shown in Additional file 1: Fig. S2D–F.

The results of the acute study showing that EV/exosome release was suppressed by DDL-112 in Thy1-αSyn mouse brain tissue (Fig. 1c) prompted us to proceed with a chronic, 5-week study of daily oral treatment of Thy1-αSyn mice with DDL-112 that included behavioral/motor analysis and determination of affects on proteinase K-resistant (PK-res) αSyn aggregation in the SN.

In the chronic study, male Thy1-αSyn mice of ~ 3 months of age received 100 mg/kg/day DDL-112 (n = 9) orally or vehicle (n = 8) for 5 weeks. During the course of the study, one mouse in each group was euthanized due to the progression of motor dysfunction that is characteristic of this model. In the last week of treatment, mice underwent behavioral/motor function assessment in Open Field (OF) [42], pole [43], and Challenging Beam (CB) [44, 45] tests. Mice were then deeply anesthetized and perfused with saline before collection of brain tissue for IHC analyses [46, 47].

After 5 weeks of treatment, the DDL-112 group made significantly (p < 0.05) fewer errors/step in the CB test than the vehicle Tg group as shown Fig. 1h. In the pole test, DDL-112 treated Thy1-αSyn took significantly less time to descend (Fig. 1i), and in OF, the distance traveled per episode of movement—indicative of the hyperactivity that is characteristic of this model [26]—was reduced for DDL-112 treated mice (Fig. 1j). Other parameters typically measured as part of these motor tests were not signficantly different between vehicle- and DDL-112-treated mice. At the end of the 5 week treatment with DDL-112, there was no significant difference in mean weight between the DDL-112 treated and vehicle groups (Fig. 1k).

The presence of PK-res αSyn aggregates in the SN of Thy1-αSyn mice is a key distinguishing characteristic of brain tissue in this model and, more importantly, in human PD with Lewy bodies [48, 49]; thus any effects of DDL-112 treatment on the levels of these aggregates [50] mediated by EV-mediated proteoapathic spread [51] was the focus of our IHC analyses. The percent area comprising PK-res αSyn aggregates in the SN globally was significantly lower (p < 0.05) in DDL-112 treated mice as shown in Fig. 1l and Additional file 1: Fig. S3A, B. Analysis of correlation between PK-res αSyn aggregates in the SN and motor assessments in CB, pole, and OF tests (Additional file 1: Fig. S3C–3H) show positive correlations that they were greater for DDL-112 treated mice in all instances. These data suggest DDL-112 treatment was able to decrease further development of pathology within the dynamic range of pathology and motor performance relationships in this model; for vehicle treated mice, pathology was beyond the dynamic range.

In the chronic study, we did not assess EV release in brain tissue due to the limitation of tissue available. EV isolation would have required too much tissue, preventing IHC analysis of αSyn aggregates. We also believe that determination of EV levels at a single time point without IL-1β stimulation of release would show only small differences between the treated and untreated groups and statistics would only be powered by use of very high n numbers.

The current in vivo studies support our previous in vitro findings [21] that the nSMase2 inhibitor, DDL-112, can suppress EV/exosome release and affect proteopathic seed propagation. The acute in vivo study shows that DDL-112 treatment results in suppression of EV release in the brain after IL-1β ICV injection, used to stimulate EV release. The chronic study demonstrated that 5-week DDL-112 treatment of Thy1-αSyn mice resulted in reduction of of PK-resistant αSyn aggregate accumulation and improved some aspects of motor function.

While our focus here is on nSMase2 inhibition by DDL-112 (cambinol), cambinol is also a known sirtuin 1 and 2 (SirT1/2) inhibitor [52], thus this mechanism and any potential effects on motor function and αSyn accumulation in the chronic study has to be considered. SirT1 has been demonstrated to affect lysosomal function and exosome secretion [53] as reported by Latifkar et al*.* who found that a reduction of SirT1 expression increased secretion of pro-tumorigenic exosomes [54]. Lee et al*.* showed that loss of SirT2 expression also increased the total number of EVs, albeit by a separate suggested mechanism than that of SirT1 [55]. Others have also reported an association between loss of SirT1 and increased EV/exosome release [56]. Based on these studies, if inhibition of SirT1 and/or 2 was implicated in treatment with DDL-112 then it could lead to increased EV release and might then be expected to exacerbate the spread of αSyn pathology, rather than ameliorate its spread as we observe in our chronic testing.

The role of SirT2 in PD is complicated. While reduction of its expression can increase EV release and potentiate disease pathology, sirtuin 2 inhibitors have been shown to block αSyn- mediated toxicity in PD models and thus could be a target for PD therapy [57, 58]. Conversely, SirT1 activation—not inhibition—has been found to be protective against αSyn-mediated toxicity, at least in cell models [59].

The potential for DDL-112 inhibition of SirT1/2 playing a role in the observed improvements in motor function and suppression of αSyn pathology is not supported by the brain levels of DDL-112 both in the acute study (Fig. 1d), as well as in the chronic study (Fig. 1m). Based on the reported inhibition potency of DDL-112 for these enzymes, the measured brain levels would likely cause a greater inhibition of brain nSMase2 enzyme activity (IC50 = 7.7 μM) [21], compared to inhibition of the enzymes SirT1 (IC50 = 56 μM) or SirT2 (IC50 = 59 μM) [60], making the role of sirtuin-mediated mechanism in DDL-112 in vivo effects unlikely.

Additional in vitro studies were performed to compare DDL-112 to potent SirT1 and SirT2 inhibitors used at 20 μM. While DDL-112 reduced EV levels, the SirT2 inhibitor increased EV release (Additional file 1: Fig. S4) providing further support to nSMase2, not SirT2, inhibition as the mechanism of action for DDL-112. The results from SirT1 inhibitor testing were inconclusive due to cell toxicity induced by the inhibitor. These findings support the likelihood that the mechanism by which DDL-112 reduced αSyn aggregates in the chronic in vivo study was due, at least in part, to inhibition of brain nSMase2 and EV release.

Others have shown that inhibition of nSMase2 decreases the transfer of oligomeric aggregates of αSyn in vitro between neurons and reduces accumulation/aggregation of high-molecular-weight α-Syn [61]. While nSMase2 inhibition has been reported to decrease tau propagation in vivo in a mouse model [20], the effects of nSMase2 inhibition on α-Syn propagation in brain in a PD model have not previously been reported.

nSMase2 is highly expressed in brain [62], with nSMase2 mRNA expression being reported to be highest in the striatum [63]. Normal phyiological levels of nSMase2 are thought to be important for protein clustering in lipid rafts. nSMase2 activity is upregulated with age [64] along with increases in long chain C24:1 ceramide levels in circulating serum EVs which can induce senescence in mesenchymal stem cells [65]. Senescence of dopaminergic neurons accompanied by a senescence-associated secretory phenotype (SASP) is suggested to be a contributing factor in the pathology of PD [66]. Increased exosome release is an integral part of SASP [67].

Mutations in the αSyn, E3 ubiquitin ligase Parkin, leucine-rich repeat kinase 2 (LRRK2), glucocerebrosidase (GBA), and acidic sphingomyelinase (SMPD1) genes—all known causes or risk factors for PD—have been linked to autophagy-lysosomal dysfunction, enhanced exosome biogenesis and exosomal αSyn load [68–73]. Manganese (Mn^2+^) exposure, an environmental risk factor of Parkinsonism, was shown to enhance αSyn-bearing exosome release, which promotes cell-to-cell propagation of pathological αSyn species including by microglia [74]. Uptake of PD-patient plasma EVs by mouse microglia cells both in vitro and in vivo results in the microglia-mediated release of αSyn-bearing exosomes, which then mediate spread of pathologic αSyn to neuronal cells [75]. Interestingly, contrary to the above examples of enhanced exosome release in PD, intracellular αSyn aggregates may increase degradation of Charged Multivesicular Body Protein 2B (CHMP2B/ESCRT-III), leading to disruption of ESCRT functions [76]. Loss-of-function mutations in ATPase cation transporting 13A2 (PARK9) also decrease intraluminal vesicle formation and exosomal release [12, 77], potentially through the ESCRT-dependent pathway. Collectively, this suggests a switch from the canonical ESCRT-dependent to a stress-induced nSMase2-dependent pathway of exosome biogenesis in PD. In agreement with this hypothesis, we demonstrate here that acute treatment with an nSMase2 inhibitor, DDL-112, affects EV release in the Tg more than in NTg mice (Additional file 1: Fig S1), decreases levels of αSyn aggregates in the SN, and improves motor functions in a PD mouse model.

Our acute in vivo study with the nSMase2 inhibitor DDL-112 shows that targeting this brain enzyme resulted in a reduction in IL-1β-mediated EV release and a trend to reduction in αSyn in the EV fraction (Additional file 1: Fig S2E). Our chronic study shows DDL-112 treatment is associated with a reduction in αSyn aggregates in the SN and improvement of motor function. These studies provide initial proof-of-concept and suggest inhibition of brain nSMase2 with molecules having improved potency and brain permeability could be a therapeutic strategy for treatment of PD. In the acute study we show that treatment with DDL-112 results in decreased EV levels and lowering of αSyn levels in EV fractions compared to vehicle, although these did not reach significance possibly due to limited animal numbers. While we did not measure EV levels in the chronic study due to the amount of tissue needed to analyse EVs, we detected DDL-112 levels in brain (Fig. 1m) that were similar to those we reported in Bilousova et al*.* [21] that were associated with inhibition of nSMase2 brain activity and thus could lead to a reduction of αSyn aggregates and improvement in motor function. In future studies, we will repeat and expand our acute IL-1β ICV mediated EV release testing paradigm to optimize the protocol for screening of additional brain permeable nSMase2 inhibitors that can decrease EV release and be used as drug candidates to suppress the spread of disease-specific proteopathic proteins.

## Supplementary Information

**Additional file 1.** Additional file.

## Data Availability

All the data generated or analysed during this study are included in the manuscript or Additional file 1.

## References

[1] Volpicelli-Daley L, Brundin P (2018). Prion-like propagation of pathology in Parkinson disease. Handb of Clin Neurol.

[2] Luk KC, Lee VMY (2014). Modeling Lewy pathology propagation in Parkinson's disease. Parkinsonism Relat Disord.

[3] Masuda-Suzukake M, Nonaka T, Hosokawa M, Kubo M, Shimozawa A, Akiyama H, Hasegawa M (2014). Pathological alpha-synuclein propagates through neural networks. Acta Neuropathol Commun.

[4] Brettschneider J, Del Tredici K, Lee VMY, Trojanowski JQ (2015). Spreading of pathology in neurodegenerative diseases: a focus on human studies. Nat Rev Neurosci.

[5] Spires-Jones TL, Attems J, Thal DR (2017). Interactions of pathological proteins in neurodegenerative diseases. Acta Neuropathol.

[6] Yamasaki TR, Holmes BB, Furman JL, Dhavale DD, Su BW, Song ES, Cairns NJ, Kotzbauer PT, Diamond MI (2019). Parkinson's disease and multiple system atrophy have distinct α-synuclein seed characteristics. J Biol Chem..

[7] Johnstone RM, Adam M, Hammond JR, Orr L, Turbide C (1987). Vesicle formation during reticulocyte maturation. Association of plasma membrane activities with released vesicles (exosomes). J Biol Chem..

[8] Théry C, Zitvogel L, Amigorena S (2002). Exosomes: composition, biogenesis and function. Nat Rev Immunol..

[9] Soria FN, Pampliega O, Bourdenx M, Meissner WG, Bezard E, Dehay B (2017). Exosomes, an Unmasked Culprit in Neurodegenerative Diseases. Front Neurosci..

[10] Alvarez-Erviti L, Seow Y, Schapira AH, Gardiner C, Sargent IL, Wood MJ, Cooper JM (2011). Lysosomal dysfunction increases exosome-mediated alpha-synuclein release and transmission. Neurobio Dis..

[11] Danzer KM, Kranich LR, Ruf WP, Cagsal-Getkin O, Winslow AR, Zhu L, Vanderburg R, McLean PJ (2012). Exosomal cell-to-cell transmission of alpha synuclein oligomers. Mol Neurodegener..

[12] Kong SM, Chan BK, Park JS, Hill KJ, Aitken JB, Cottle L, Farghaian H, Cole AR, Lay PA, Sue CM, Cooper AA (2014). Parkinson's disease-linked human PARK9/ATP13A2 maintains zinc homeostasis and promotes alpha-Synuclein externalization via exosomes. Hum Mol Genet..

[13] Stuendl A, Kunadt M, Kruse N, Bartels C, Moebius W, Danzer KM, Mollenhauer B, Schneider A (2016). Induction of alpha-synuclein aggregate formation by CSF exosomes from patients with Parkinson's disease and dementia with Lewy bodies. Brain..

[14] Gustafsson G, Loov C, Persson E, Lazaro DF, Takeda S, Bergstrom J, Erlandsson A, Sehlin D, Balaj L, Gyorgy B, Hallbeck M, Outeiro TF, Breakefield XO, Hyman BT, Ingelsson M (2018). Secretion and Uptake of alpha-Synuclein Via Extracellular Vesicles in Cultured Cells. Cell Mol Neurobiol..

[15] Ngolab J, Trinh I, Rockenstein E, Mante M, Florio J, Trejo M, Masliah D, Adame A, Masliah E, Rissman RA (2017). Brain-derived exosomes from dementia with Lewy bodies propagate alpha-synuclein pathology. Acta Neuropathol Commun..

[16] Emmanouilidou E, Melachroinou K, Roumeliotis T, Garbis SD, Ntzouni M, Margaritis LH, Stefanis L, Vekrellis K (2010). Cell-produced alpha-synuclein is secreted in a calcium-dependent manner by exosomes and impacts neuronal survival. J Neurosci..

[17] Trajkovic K, Hsu C, Chiantia S, Rajendran L, Wenzel D, Wieland F, Schwille P, Brugger B, Simons M (2008). Ceramide triggers budding of exosome vesicles into multivesicular endosomes. Science..

[18] Lim YJ, Lee SJ (2017). Are exosomes the vehicle for protein aggregate propagation in neurodegenerative diseases?. Acta Neuropathol Commun..

[19] Dinkins MB, Dasgupta S, Wang G, Zhu G, Bieberich E (2014). Exosome reduction in vivo is associated with lower amyloid plaque load in the 5XFAD mouse model of Alzheimer's disease. Neurobiol Aging..

[20] Asai H, Ikezu S, Tsunoda S, Medalla M, Luebke J, Haydar T, Wolozin B, Butovsky O, Kugler S, Ikezu T (2015). Depletion of microglia and inhibition of exosome synthesis halt tau propagation. Nat Neurosci..

[21] Bilousova T, Elias C, Miyoshi E, Alam MP, Zhu C, Campagna J, Vadivel K, Jagodzinska B, Gylys KH, John V (2018). Suppression of tau propagation using an inhibitor that targets the DK-switch of nSMase2. Biochem Biophys Res Commun..

[22] Figuera-Losada M, Stathis M, Dorskind JM, Thomas AG, Bandaru VV, Yoo SW, Westwood NJ, Rogers GW, McArthur JC, Haugley NJ, Slusher BS, Rojas C (2015). Cambinol, a novel inhibitor of neutral sphingomyelinase 2 shows neuroprotective properties. PloS One..

[23] Menck K, Sonmezer C, Worst TS, Schulz M, Dihazi GH, Streit F, Erdmann G, Kling S, Boutros M, Binder C, Gross JC (2017). Neutral sphingomyelinases control extracellular vesicles budding from the plasma membrane. J Extracell Vesicles..

[24] Filippov V, Song MA, Zhang K, Vinters HV, Tung S, Kirsch WM, Yang J, Duerksen-Hughes PJ (2012). Increased ceramide in brains with Alzheimer's and other neurodegenerative diseases. J Alzheimers Dis..

[25] Mielke MM, Bandaru VV, Haughey NJ, Rabins PV, Lyketsos CG, Carlson MC (2010). Serum sphingomyelins and ceramides are early predictors of memory impairment. Neurobiol Aging..

[26] Chesselet MF, Richter F, Zhu C, Magen I, Watson MB, Subramaniam SR (2012). A progressive mouse model of Parkinson's disease: the Thy1-aSyn mice. Neurotherapeutics..

[27] Rockenstein E, Mallory M, Hashimoto M, Song D, Shults CW, Lang I, Masliah E (2002). Differential neuropathological alterations in transgenic mice expressing alpha-synuclein from the platelet-derived growth factor and Thy-1 promoters. J Neurosci Res..

[28] Fleming SM, Salcedo J, Fernagut PO, Rockenstein E, Masliah E, Levine MS, Chesselet MF (2004). Early and progressive sensorimotor anomalies in mice overexpressing wild-type human alpha-synuclein. J Neurosci..

[29] Bilousova T, Simmons BJ, Knapp RR, Elias CJ, Campagna J, Melnik M, Chandra S, Fotch S, Zhu C, Vadivel K, Jagodzinska B, Cohn W, Spilman P, Gylys KH, Garg N, John V (2020). Dual Neutral Sphingomyelinase-2/Acetylcholinesterase Inhibitors for the Treatment of Alzheimer's Disease. ACS Chem Biol..

[30] Huang Y, Cheng L, Turchinovich A, Mahairaki V, Troncoso JC, Pletnikova O, Haughey NJ, Vella LJ, Hill AF, Zheng L, Witwer KW (2020). Influence of species and processing parameters on recovery and content of brain tissue-derived extracellular vesicles. J Extracell Vesicles..

[31] Vella LJ, Scicluna BJ, Cheng L, Bawden EG, Masters CL, Ang CS, Williamson N, Mclean C, Barnham K, Hill AF (2017). A rigorous method to enrich for exosomes from brain tissue. J Extracell Vesicles..

[32] Vassileff N, Vella LJ, Rajapaksha H, Shambrook M, Kenari AN, McLean C, Hill AF, Cheng L (2020). Revealing the proteome of motor cortex derived extracellular vesicles isolated from amyotrophic lateral sclerosis human postmortem tissues. Cells..

[33] Bosch S, de Beaurepaire L, Allard M, Mosser M, Heichette C, Chretien D, Jegou D, Bach JM (2016). Trehalose prevents aggregation of exosomes and cryodamage. Sci Rep..

[34] Nalivaeva NN, Rybakina EG, Pivanovich I, Kozinets IA, Shanin SN, Bartfai T (2000). Activation of neutral sphingomyelinase by IL-1beta requires the type 1 interleukin 1 receptor. Cytokine..

[35] Rutkute K, Asmis RH, Nikolova-Karakashian MN (2007). Regulation of neutral sphingomyelinase-2 by GSH: a new insight to the role of oxidative stress in aging-associated inflammation. J Lipid Res..

[36] Cypryk W, Nyman TA, Matikainen S (2018). From inflammasome to exosome-does extracellular vesicle secretion constitute an inflammasome-dependent immune response?. Front Immunol..

[37] Rojas C, Barnaeva E, Thomas AG, Hu X, Southall N, Marugan J, Chaudri AD, Yoo SW, Hin N, Stepanek O, Wu Y, Zimmermann SC, Gadiano AG, Tsukamoto T, Rais R, Haughey N, Ferrer M, Slusher BS (2018). DPTIP, a newly identified potent brain penetrant neutral sphingomyelinase 2 inhibitor, regulates astrocyte-peripheral immune communication following brain inflammation. Sci Rep..

[38] Schulte T, Schöls L, Müller T, Woitalla D, Berger K, Krüger R (2002). Polymorphisms in the interleukin-1 alpha and beta genes and the risk for Parkinson's disease. Neurosci Lett..

[39] Koprich JB, Reske-Nielsen C, Mithal P, Isacson O (2008). Neuroinflammation mediated by IL-1beta increases susceptibility of dopamine neurons to degeneration in an animal model of Parkinson's disease. J Neuroinflammation..

[40] Leal M, Casabona J, Puntel M, Pitossi F (2013). Interleukin-1beta and TNF-alpha: Reliable targets for protective therapies in Parkinson's disease?. Front Cell Neurosci..

[41] Baietti MF, Zhang Z, Mortier E, Melchior A, Degeest G, Geeraerts A, Ivarsson Y, Depoortere F, Coomas C, Vermeiren E, Zimmerman P, David G (2012). Syndecan-syntenin-ALIX regulates the biogenesis of exosomes. Nat Cell Biol..

[42] Seibenhener ML, Wooten MC. Use of the Open Field Maze to measure locomotor and anxiety-like behavior in mice. J Vis Exp. 2015;(96):e52434-e. 10.3791/52434 PMC4354627 25742564

[43] Brooks SP, Dunnett SB (2009). Tests to assess motor phenotype in mice: a user's guide. Nat Rev Neurosci..

[44] Richter F, Gao F, Medvedeva V, Lee P, Bove N, Fleming SM, Michaud M, Lemerse V, Patassini S, De La Rosa K, Mulligan CK, Sioshani PC, Zhu C, Coppola G, Bordet T, Pruss RM, Chesselet MF (2014). Chronic administration of cholesterol oximes in mice increases transcription of cytoprotective genes and improves transcriptome alterations induced by alpha-synuclein overexpression in nigrostriatal dopaminergic neurons. Neurobiol Dis..

[45] Fleming SM, Salcedo J, Fernagut PO, Rockenstein E, Masliah E, Levine MS, Chesselet MF (2004). Early and progressive sensorimotor anomalies in mice overexpressing wild-type human alpha-synuclein. J Neurosci..

[46] Richter F, Subramaniam SR, Magen I, Lee P, Hayes J, Attar A, Zhu C, Frainch F, Bove N, De La Rosa K, Kwong J, Klarner FG, Schrader T, Chesselet MF, Bitan G (2017). A molecular tweezer ameliorates motor deficits in mice overexpressing α-synuclein. Neurotherapeutics..

[47] Subramaniam SR, Magen I, Bove N, Zhu C, Lemesre V, Dutta G, Elias CJ, Lester HA, Chesselet MF (2018). Chronic nicotine improves cognitive and social impairment in mice overexpressing wild type α-synuclein. Neurobiol Dis..

[48] Neumann M, Müller V, Kretzschmar HA, Haass C, Kahle PJ (2004). Regional distribution of proteinase K-resistant α-synuclein correlates with lewy body disease stage. J Neuropathol Exp Neurol..

[49] Poewe W, Seppi K, Tanner CM, Halliday GM, Brundin P, Volkmann J, Schrag AE, Lang AE (2017). Parkinson disease. Nat Rev Dis Primers..

[50] Candelise N, Schmitz M, Llorens F, Villar-Piqué A, Cramm M, Thom T, Margarida da Silva Correia S, Gomes da Cunha JE, Mobius W, Outeiro TF, Alvarez VG, Banchelli M, D'Andrea C, de Angelis M, Zafar S, Rabano A, Matteini P, Zerr I. Seeding variability of different alpha synuclein strains in synucleinopathies. Ann Neurol. 2019;85(5):691–703. 10.1002/ana.25446 30805957

[51] Vella LJ, Hill AF, Cheng L (2016). Focus on extracellular vesicles: exosomes and their role in protein trafficking and biomarker potential in Alzheimer's and Parkinson's disease. Int J Mol Sci..

[52] Villalba JM, Alcain FJ (2012). Sirtuin activators and inhibitors. Biofactors..

[53] McAndrews KM, LeBleu VS, Kalluri R (2019). SIRT1 regulates lysosome function and exosome secretion. Dev Cell..

[54] Latifkar A, Ling L, Hingorani A, Johansen E, Clement A, Zhang X, Hartman J, Fischbach C, Lin H, Cerione RA, Antonyak MA (2019). Loss of sirtuin 1 alters the secretome of breast cancer cells by impairing lysosomal integrity. Dev Cell..

[55] Lee BR, Sanstrum BJ, Liu Y, Kwon SH (2019). Distinct role of Sirtuin 1 (SIRT1) and Sirtuin 2 (SIRT2) in inhibiting cargo-loading and release of extracellular vesicles. Sci Rep..

[56] Han L, Long Q, Li S, Xu Q, Zhang B, Dou X, Qian M, Jiramongkol Y, Guo J, Chin YE, Lam EWF, Jiang J, Sun Y (2020). Senescent stromal cells promote cancer resistance through SIRT1 loss-potentiated overproduction of small extracellular vesicles. Cancer Res..

[57] Liu Y, Zhang Y, Zhu K, Chi S, Wang C, Xie A (2019). Emerging role of Sirtuin 2 in Parkinson's disease. Front Aging Neurosci..

[58] Outeiro TF, Kontopoulos E, Altmann SM, Kufareva I, Strathearn KE, Amore AM, Volk CB, Maxwell MM, Rochet JC, McLean PJ, Young AB, Abagyan R, Feany MB, Hyman BT, Kazantsev AG (2007). Sirtuin 2 inhibitors rescue alpha-synuclein-mediated toxicity in models of Parkinson's disease. Science..

[59] Albani D, Polito L, Batelli S, De Mauro S, Fracasso C, Martelli G, Colombo L, Manzoni C, Salmona M, Caccia S, Negro A, Forloni G (2009). The SIRT1 activator resveratrol protects SK-N-BE cells from oxidative stress and against toxicity caused by alpha-synuclein or amyloid-beta (1–42) peptide. J Neurochem..

[60] Lugrin J, Ciarlo E, Santos A, Grandmaison G, dos Santos I, Le Roy D, Roger T (2013). The sirtuin inhibitor cambinol impairs MAPK signaling, inhibits inflammatory and innate immune responses and protects from septic shock. Biochim Biophys Acta..

[61] Sackmann V, Sinha MS, Sackmann C, Civitelli L, Bergström J, Ansell-Schultz A, Hallbeck M. Inhibition of nSMase2 reduces the transfer of oligomeric α-synuclein irrespective of hypoxia. Front Mol Neurosci. 2019;12(200). 10.3389/fnmol.2019.00200 PMC6724746 31555088

[62] Hofmann K, Tomiuk S, Wolff G, Stoffel W (2000). Cloning and characterization of the mammalian brain-specific, Mg2+-dependent neutral sphingomyelinase. Proc Natl Acad Sci U S A..

[63] Tan LH, Tan AJ, Ng YY, Chua JJ, Chew WS, Muralidharan S, Torta F, Dutta B, Sze SK, Herr D, Ong WY (2018). Enriched Expression of Neutral Sphingomyelinase 2 in the Striatum is Essential for Regulation of Lipid Raft Content and Motor Coordination. Mol Neurobiol..

[64] Crivello NA, Rosenberg IH, Dallal GE, Bielinski D, Joseph JA (2005). Age-related changes in neutral sphingomyelin-specific phospholipase C activity in striatum, hippocampus, and frontal cortex: implication for sensitivity to stress and inflammation. Neurochem Int..

[65] Khayrullin A, Krishnan P, Martinez-Nater L, Mendhe B, Fulzele S, Liu Y, Mattison JA, Hamrick MW (2019). Very long-chain C24:1 ceramide is increased in serum extracellular vesicles with aging and can induce senescence in bone-derived mesenchymal stem cells. Cells..

[66] Riessland M, Kolisnyk B, Kim TW, Cheng J, Ni J, Pearson JA, Park EJ, Dam K, Acehan D, Ramos-Espiritu LS, Wang W, Zhang J, Shim JW, Ciceri G, Brichta L, Studer L, Greengard P. Loss of SATB1 Induces p21-Dependent Cellular Senescence in Post-mitotic Dopaminergic Neurons. Cell Stem Cell. 2019;25(4):514–30 e8. 10.1016/j.stem.2019.08.013 PMC7493192 31543366

[67] Jakhar R, Crasta K (2019). Exosomes as emerging pro-tumorigenic mediators of the senescence-associated secretory phenotype. Int J Mol Sci..

[68] Tofaris GK (2017). A critical assessment of exosomes in the pathogenesis and stratification of Parkinson's disease. J Parkinsons Dis..

[69] Tofaris GK, Goedert M, Spillantini MG (2017). The transcellular propagation and intracellular trafficking of alpha-synuclein. Cold Spring Harbor Perspect Med..

[70] Papadopoulos VE, Nikolopoulou G, Antoniadou I, Karachaliou A, Arianoglou G, Emmanouilidou E, Sardi SP, Stefanis L, Vekrellias K (2018). Modulation of beta-glucocerebrosidase increases alpha-synuclein secretion and exosome release in mouse models of Parkinson's disease. Hum Mol Genet..

[71] Cerri S, Ghezzi C, Sampieri M, Siani F, Avenali M, Dornini G, Zangaglia R, Minafra B, Blandini F (2018). The exosomal/total alpha-synuclein ratio in plasma is associated with glucocerebrosidase activity and correlates with measures of disease severity in PD patients. Front Cell Neurosci..

[72] Bae EJ, Kim DK, Kim C, Mante M, Adame A, Rockenstein E, Ulusoy A, Klinkenberg M, Jeong GR, Bae JR, Lee C, Lee HJ, Lee BD, Di Monte AD, Masliah E, Lee SJ (2018). LRRK2 kinase regulates alpha-synuclein propagation via RAB35 phosphorylation. Nat Commun..

[73] Alcalay RN, Mallett V, Vanderperre B, Tavassoly O, Dauvilliers Y, Wu RYJ, Ruskey JA, Leblond CS, Ambalvanan A, Laurent SB, Spiegelman D, Dionne-Laporte A, Liong C, Levy OA, Fahn S, Waters C, Kuo SH, Chung WK, Ford B, Marder KS, Kang UJ, Hassin-Baer S, Greenbaum L, Trempe JF, Wolf P, Oliva P, Zhang XK, Clark LN, Langlois M, Dion PA, Fon EA, Dupre N, Rouleau GA, Gan-Or Z (2019). SMPD1 mutations, activity, and alpha-synuclein accumulation in Parkinson's disease. Mov Disord..

[74] Harischandra DS, Rokad D, Neal ML, Ghaisas S, Manne S, Sarkar S, Panicker N, Zenitsky G, Jin H, Lewis M, Huang X, Anantharam V, Kanthasamy A, Kanthasamy AG (2019). Manganese promotes the aggregation and prion-like cell-to-cell exosomal transmission of alpha-synuclein. Sci Signal..

[75] Xia Y, Zhang G, Han C, Ma K, Guo X, Wan F, Kou L, Yin S, Liu L, Huang J, Xiong N, Wang T (2019). Microglia as modulators of exosomal alpha-synuclein transmission. Cell Death Dis..

[76] Spencer B, Kim C, Gonzalez T, Bisquertt A, Patrick C, Rockenstein E, Adame A, Lee SJ, Desplats P, Masaliah E (2016). alpha-Synuclein interferes with the ESCRT-III complex contributing to the pathogenesis of Lewy body disease. Hum Mol Genet..

[77] Tsunemi T, Hamada K, Krainc D (2014). ATP13A2/PARK9 regulates secretion of exosomes and alpha-synuclein. J Neurosci..

