# Correction to: Pharmacological inhibition of nSMase2 reduces brain exosome release and α-synuclein pathology in a Parkinson’s disease model

**DOI:** 10.1186/s13041-021-00816-4

**Published:** 2021-07-08

**Authors:** Chunni Zhu, Tina Bilousova, Samantha Focht, Michael Jun, Chris Jean Elias, Mikhail Melnik, Sujyoti Chandra, Jesus Campagna, Whitaker Cohn, Asa Hatami, Patricia Spilman, Karen Hoppens Gylys, Varghese John

**Affiliations:** 1 Drug Discovery Lab, Department of Neurology, University of California, Los Angeles, CA 90095 USA; 2 School of Nursing, University of California, Los Angeles, CA 90095 USA

## Correction to: Mol Brain (2021) 14:70 https://doi.org/10.1186/s13041-021-00776-9

Following publication of the original article [1], it was noted that due to a typesetting mistake, an incorrect version of Additional file 1 (with regards to the reference numbering) was processed.

The correct version of Additional file 1 is attached to this Correction and has been corrected in the original article. The publisher apologises to the authors and readers for the inconvenience caused by this error.

## Supplementary Information

**Additional file 1.** Additional file.

## References

[1] Zhu C, Bilousova T, Focht S, Jun M, Elias CJ, Melnik M, Chandra S, Campagna J, Cohn W, Hatami A, Spilman P, Gylys KH, John V (2021). Pharmacological inhibition of nSMase2 reduces brain exosome release and α-synuclein pathology in a Parkinson’s disease model. Mol Brain.

