# Supplementary material for: Correction to: Pharmacological inhibition of nSMase2 reduces brain exosome release and α-synuclein pathology in a Parkinson’s disease model
Source: Mol Brain. 2021 Jul 8;14:108. doi: 10.1186/s13041-021-00816-4 (PMC8265003; doi:10.1186/s13041-021-00816-4)
Supplement: Supplementary file 1 — Additional file 1. Additional file. [file 13041_2021_816_MOESM1_ESM.pdf]

Pharmacological inhibition of nSMase2 reduces brain exosome release and α-synuclein pathology in a Parkinson's disease model

Chunni Zhu[1‡], Tina Bilousova[1,2‡], Samantha Focht[1], Michael Jun[1], Chris Jean Elias[1], Mikhail Melnik[1], Sujyoti Chandra[1], Jesus Campagna[1], Whitaker Cohn[1], Asa Hatami[1], Patricia Spilman[1], Karen Gylys[2], Varghese John[1*]

[1]Drug Discovery Lab, Department of Neurology, University of California, Los Angeles, CA 90095, USA

[2]School of Nursing, University of California, Los Angeles, CA 90095, USA

[‡]These authors contributed equally to the manuscript

*Corresponding author: Prof. Varghese John; Phone: (310) 206-4345; Fax: (310) 825-6956; Email: VJohn@mednet.ucla.edu

Additional File 1

Materials and Methods

Animals

Animal care was performed in accordance with the United States Public Health Service Guide for the Care and Use of Laboratory Animals, with approval to the Drug Discovery Lab by the Institutional Animal Care and Use Committee at the University of California Los Angeles (UCLA). Male transgenic mice overexpressing human wildtype α-synuclein under control of the Thy-1 promoter (Thy1-αSyn) (27) maintained on a hybrid C57BL/6-DBA/2 background (28) were used in this study. The genotype of all Thy1-αSyn mice was determined by polymerase chain reaction (PCR) amplification analysis of tail DNA at three weeks of age and verified at the end of the experiment. Mice enrollment in the study balanced litters across treatment groups. Investigators involved in drug administration, data gathering, and data analyses were unaware of genotype and treatment. Animals were maintained on a reverse light/dark cycle with lights off at 10 am, and all testing was performed between 1- 4 pm during the dark cycle under low light. Food and water was available *ad libitum*.

In vitro comparison of DDL-112 to SirT1 and SirT2 inhibitors

The effects of DDL-112, AGK2 (a SirT1 inhibitor), and EX-527 (aSirT2 inhibitor) on exosomal αSyn levels was evaluated *in vitro*. HEK-293T αSyn A53T FRET biosensor (αSyn biosensor) cells were grown in Dulbecco′s Modified Eagle′s medium (DMEM) with high glucose, 10% FBS, and 1% penicillin-streptomycin at 37°C/5% CO_2 (6). The cells were seeded with αSyn-PFF (type 1, StressMarq Bioscience, Canada) using transfection reagent lipofectamine 2000 (Thermofisher Scientific, MA); control cells were treated with a corresponding amount of the

transfection reagent without αSyn-PFF. After 24-hr incubation, the cells were trypsinized and plated in presence of test compounds or DMSO for 48 hrs. Seeding efficacy was confirmed using Lionheart FX imaging system equiped with CFP/YFP FRET filter cube and 4x objective (BioTek, VT). A small sample of medium was collected for lactate dehydrogenase (LDH) assay (Promega, WI) and the rest of the medium was used for EV purification with ExoQuick-TC reagent (System Biosciences, CA) according to manufacturer's instructions after 48-hr incubation with compounds.

Preliminary in vivo study

In vivo effects of DDL-112 treatment on EV release and EV exosomal marker CD63, syntenin-1(Synt-1), calnexin (CNX), and α-synuclein (αSyn) to assist with design of subsequent acute and chronic studies, male 4-5 mo Thy1-αSyn transgenic (Tg) or non-transgenic littermate mice were either treated with DDL-112 orally at 100 mg/kg or vehicle-only (DMSO). For the Tg DDL-112 and vehicle groups, N = 6, and for the NTg groups, N = 3. All mice were euthanized 3 hours after dosing by over-anesthesia and saline perfusion, and brain tissue collected for determination of DDL-112 brain levels and EV/exosome purification and analysis.

Determination of DDL-112 brain levels

Analysis of plasma and brain concentrations was done using a targeted liquid chromatography-tandem mass spectrometry (LC-MS/MS) assay developed for DDL-112 using the multiple reaction monitoring (MRM) acquisition method on a 6460 triple quadrupole mass spectrometer (Agilent Technologies) coupled to a 1290 Infinity HPLC system (Agilent Technologies) with a Phenomenex analytical column (Kinetex 1.7 µm C18 100 Å 100 x 2.1 mm). The HPLC method utilized a mixture of solvent A (99.9/1 water/formic acid) and solvent B (99.9/1

acetonitrile/aormic Acid) and a gradient was use for the elution of the compounds (min/%B: 0/20, 5/20, 20/99, 22/99, 25/20, 35/20).

In this assay, detection of fragment ions originating from cambinol (m/z: 361.2 to 217.1, 158.1) as well as LC retention time (RT= 14.9 min) were utilized to ensure compound specificity and accurate quantification in the biological samples. An internal standard similar to DDL-112 (m/z: 256.1; 10 pmol; RT = 10.3) was added to each sample. Standards were made in drug naïve brain lysates with increasing amounts of DDL-112 (S1,S2: 0 pmol/ S3,S4: 1 pmol/ S5,S6: 10 pmol/ S7,S8: 100 pmol, S9,S10: 1000 pmol). A standard curve was made by plotting the amount of DDL-112 per standard vs. the ratio of measured chromatographic peak areas for each compound (DDL-112/internal standard). The trendline equation was then used to calculate the absolute concentrations of cambinol in brain.

EV/exosome protein analysis

EV/exosomes were collected, purified, and protein content analyzed as described below for the acute study.

Acute EV Release Study

DDL-112 pretreatment and IL-1β ICV injection

Thy1-αSyn mice were dosed with DDL-112 (Abcam, Cambridge, MA) at 100 mg/kg by oral gavage; this is estimated to result in mean ~0.375 µM of DDL-112 in the brain (21). One hour later, mice were deeply anesthetized and received 0.2 ng IL-1β (LifeSpan BioSciences, Seattle, WA) by unilateral ICV injection (0.2 mm posterior from bregma, 1 mm to the right from midline, at a depth 1.8 mm); 2 hours later, mice were perfused/euthanized and brain tissue was collected

for EV isolation and analysis; cerebellum was used to determine DDL-112 tissue levels. There were 4 mice per group and 3 groups: control (vehicle ICV injection), IL-1β ICV only, and DDL-112 pretreatment with IL-1β ICV.

Isolation of brain EVs, TEM, TRPS and immunoblotting

Brains (minus cerebellum) were weighed, minced in ice-cold Hibernate-A medium (ThermoFisher Scientific, A1247501 Waltham, MA) and tissue was gently dissociated using an adult brain dissociation kit and GentleMACS dissociator according to manufactirer's instructions (Miltenyi Biotec, San Diego, CA). EV fractions were purified by sequential differential and sucrose gradient rate-zonal ultracentrifugation, followed by a washing step as previously described (29-32), Briefly, cells and debris were pelleted in three sequential centrifugations (300 x g for 10 min, 2000 x g for 10 min, and 10,000 x g for 30 min). Supernatant from the last centrifugation was applied to a triple sucrose cushion (2.5 M,1.3 M, and 0.6 M sucrose in 20 mM HEPES, pH 7.4) and ultracentrifugation (180,000 x g for 3 hrs, swinging bucket rotor) was performed to separate vesicles based on the density; three fractions (F1, F2, and F3) were collected for initial protocol validation. For most of the brain samples only F2: EV/exosomal fraction was collected for further analysis. EV/exosomes were pelleted/washed in 50 ml of PBS (100,000 x g for 1 hr 15 min) and EV-containing pellets were resuspended in cryopreservation solution (25 mM trehalose in PBS, pH 7.4) with protease and phosphatase inhibitor cocktail and frozen at -80°C (33). The volume of cryopreservation solution for each sample was calculated based on weight so that the brain sample used for EV isolation was 0.4 g of tissue/150 µl solution.

For transmission electron microscopy (TEM) analysis, small amounts of purified EVs were fixed on a copper mesh in glutaraldehyde/paraformaldehyde solution, stained with 2% uranyl

acetate solution and imaged on a JEOL 100CX electron microscope (Jeol USA, Peabody, MA) at 29K X magnification.

Samples of EVs also underwent Tunable Resistive Pulse Sensing (TRPS) analysis using qNano Gold instrument with NP100 nanopores (Izon Science, Medford, MA).

Proteins from EV fractions were separated by 10–20% Tris-Glycine SDS-PAGE under non-reducing conditions, transferred to PVDF membrane, labeled with Ponceau S solution (0.1 % w/v in 5% acetic acid) for protein loading control, and probed with antibodies against exosomal markers CD63 (ThermoFisher Scientific, 10628D, Waltham, MA), syntenin-1 (SantaCruz Biotech, sc-48742, Dallas, TX), GM-130 (Novus Biologicals, NBP2-53420SS, Centennial, CO), calnexin (SantaCruz Biotech, sc-23954, Dallas, TX), and human αSyn (BioLegend, Clone 4B12, San Diego, CA), followed by HRP-conjugated secondary antibodies (Jackson Immunoresearch Labs, West Grove, PA). Chemiluminescent signals were obtained with Super Signal West Femto substrate (Thermo Fisher Scientific, Waltham, MA), detected using a BioSpectrum 600 imaging system, and quantified using VisionWorks Version 6.6A software (UVP; Upland, CA).

DDL-112 brain levels were determined as described above for the Preliminary Study.

Chronic 5-week *in vivo* study

Treatment

We treated Thy1-αSyn mice with either DDL-112 at 100 mg/kg/day (mkd) or vehicle only (Veh) by oral gavage for 35 days (5-weeks). The stock solution used for gavage was 50 mg/ml DDL-112 in 95% corn oil/5% ethanol. At the start of the study, there were 9 mice in the Thy1-αSyn Veh group and 8 mice in the DDL-112 group. Animals were all 3 months +/- 2 weeks of age

at the start of treatment. Animal weights were recorded pre-study, twice weekly during dosing, and on the last day of dosing before euthanasia.

Behavioral studies

Open field (OF): spontaneous activity was measured in an Open Field (25.5 cm × 25.5 cm) for 15 min using an automated system (Truscan system for mice; Coulbourn Instruments, Allentown, PA) at the end of the treatment, before euthanasia. Mice also underwent pole and challenging beam tests of motor function at the end of the treatment, before euthanasia following previously published protocols (45).

Euthanasia and tissue collection

Mice were euthanized by pentobarbitol over-anesthesia and cold saline perfusion 4 hours after dosing on the last day of the study. Whole brains were removed and halved down the mid-line. One half hemisphere was immersion fixed in 4% paraformaldehyde for 72 hrs, cyroprotected in 30% sucrose, and then frozen in powdered dry ice. Tissues were stored at -80°C before cryosectioning. The other half was saved unfixed for further studies.

Immunohistochemical (IHC) analysis of Proteinase K (PK) resistant αSyn

Coronal brain sections at 40 µm were sectioned on cryostat (Leica CM 1850, IL) and stored in cryoprotectant at -20°C. Sections at Bregma -3.52 mm from the Thy1-αSyn hemizygous mice were selected for αSyn aggregate quantification in the substantia nigra (SN) as previously described (46, 47). Sections were washed in phosphate buffered saline (PBS, pH 7.4), incubated with 5 µg/ml proteinase K (Invitrogen, Carlsbad, CA) for 10 min. Endogenous peroxidase activity was blocked with 0.5% H_2O_2 in PBS for 15 min. Nonspecific binding was blocked with mouse

IgG blocking reagent M.O.M kit (Vector Laboratories, Burlingame, CA) for 1 hour at room temperature. Sections were incubated with mouse anti-αSyn (1:250, Cat # 610787, BD Biosciences, San Jose, CA) at 4°C overnight. After washing in PBS, sections were incubated with secondary antibody biotinylated goat anti-mouse IgG (1:200, Vector Laboratories, Burlingame, CA) for 2 hours. The avidin-biotin complex method was used to detect the secondary antibody (ABC elite kit, Vector laboratories, Burlingame, CA) and the reaction product was visualized by 3,3'-diaminobenzidine tetrachloride (DAB, Sigma, St Louis, MO). Control sections are incubated with mouse IgG1 (1 µg/ml, Sigma). Sections were dehydrated and cleared with xylene, mounted with Eukitt mounting medium (Calibrated Instruments, Hawthorne, NY), and examined under bright-field illumination with a Zeiss Axioskop microscope (Zeiss, Thornwood, NY). Digital images were captured by a Spot digital camera (Spot Imaging, Sterling Heights, MI).

Quantification of PK-resistant αSyn Aggregates in the SN

Images were acquired from the substantia nigra (SN) using a Leica DM-LB microscope with a Ludi XYZ motorized stage and z-axis microcator (MT12, Heidenheim, Traunreut, Germany) and the StereoInvestigator software (MicroBrightField, Colchester, VT). First, contours were drawn to delineate the SN at 5X magnification for anatomical accuracy. Then, images were acquired at 40X magnification by positioning the camera at the desired location to attain images with consistent lighting and color balance.

An investigator blinded to genotype and treatment analyzed the acquired images with the ImageJ software (NIH) by first transforming the images to 8-bit files. The aggregates were then overexposed by adjusting the brightness/contrast and a threshold was visually determined to confirm the particles. The scale was set to 3.79 pixels/µm and the particles were measured if they

were between 0.5-50 μm² and had circularities between 0.3-0.9. Total surface area of the labeled aggregates was calculated for each image and used for further analysis.

Statistical analysis

One-way analysis of variance (ANOVA) was used for multiple comparisons with post-hoc Tukey's HSD test (the Acute study with 3 groups) or Student's t-test (chronic study with 2 groups) for direct comparison of Veh and DDL-112-treated transgenic Thy1-αSyn mice. The level of significance was set at $p < 0.05$* (<0.01 **; <0.001***; <0.0001****). All statistical analysis was performed with GraphPad Prism software.

Additional Figures

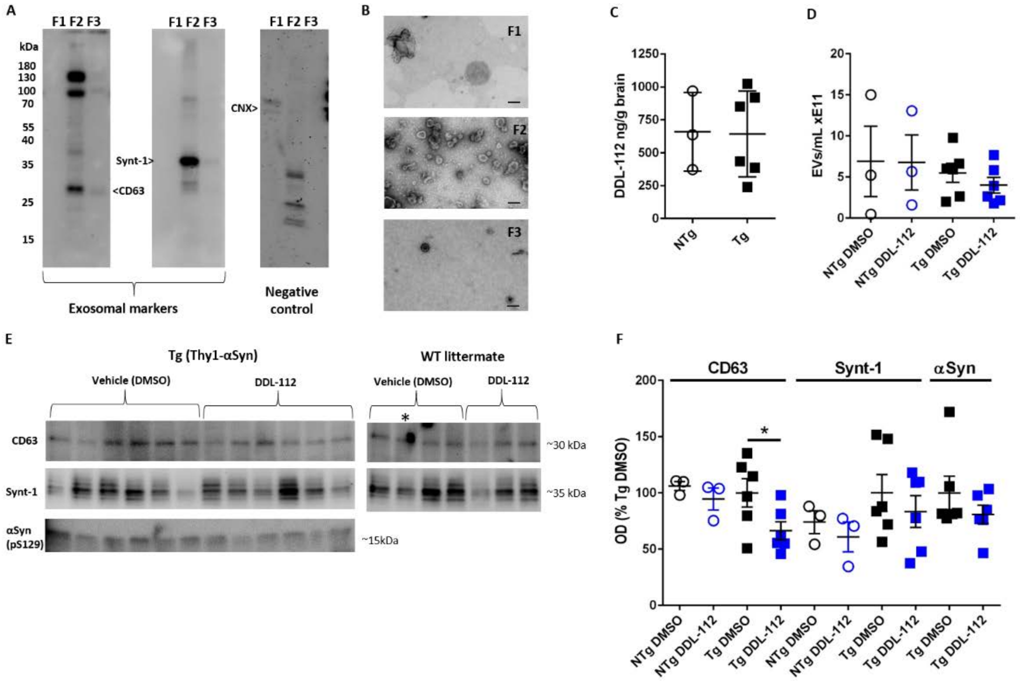

Fig. S1. *Validation of exosome purification protocol and preliminary study of DDL-112 effect on exosome release in Tg mice and NTg littermates*. (A) Immunoblot (IB) analyses of proteins enriched in exosomes, CD63 and Syntenin-1 (Synt-1) and negative control marker, endoplasmic reticulum protein calnexin (CNX) F1, F2, and F3 fractions collected from sucrose gradient. Equal volumes of each fraction (24 µl) were loaded into the gel. (B) Representative transmission electron microscopy (TEM) images of F1, F2, and F3 fractions (bar size is 100 nm). (C) Brain levels of DDL-112 in Tg and NTg mice 3 hrs after oral dosing at 100 mg/kg. (D) Average concentrations of 50-200 nm size EVs from Tg and NTg mice treated with DDL-112 or vehicle (DMSO) are shown. (E) Images of immunoblot (IB) analysis of EV fractions from individual animals probed with CD63, Synt-1, and α-synuclein antibodies. A band artifact in one of NTg/DMSO samples at the CD63 IB ($p<0.05$) led to exclusion of that sample from all the analysis. (F) Densitometry analysis of the IB images is shown. Optical density (OD) is shown as percent of Tg group treated with DMSO. To compare Veh Tg to DDL-112 Tg, an unpaired Student's t-test was used ($p = 0.0487$). N = 6 animals per group for Tg mice and N = 3 animals per group for NTg littermates.

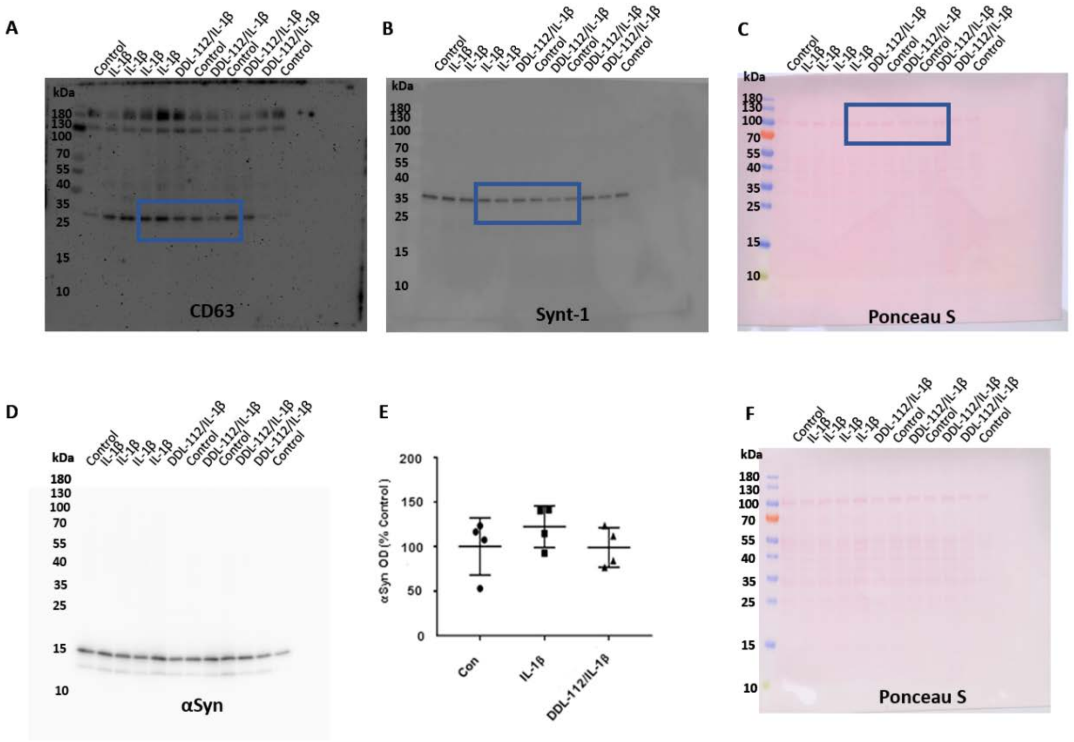

Fig. S2. *Acute Study EV fraction CD63, Synt-1, αSyn, and respective Ponceau S stained membranes.* (A) A representative image of an immunoblot of EV fractions probed with antibodies against CD63 (full membrane). (B) A representative image of an immunoblot of EV fractions probed with antibodies against Syntenin-1 (full membrane, stripped and re-probed after CD63 immunolabeling). (C) Respective membrane (immunoblot shown in A and B) stained with Ponceau S to confirm equal loading of samples and quality of transfer. Blue boxes demonstrate areas of the immunoblots presented in Fig. 1E. (D) A representative image of an immunoblot of EV fractions probed with antibodies against human αSyn (Biolegend, Clone 4B12) is shown. (E) The band densitometry analysis, plotted as OD percentage of control for the immunoblots is graphed (n = 4 animals per group). (F) The Ponceau S stained blot used as a loading control.

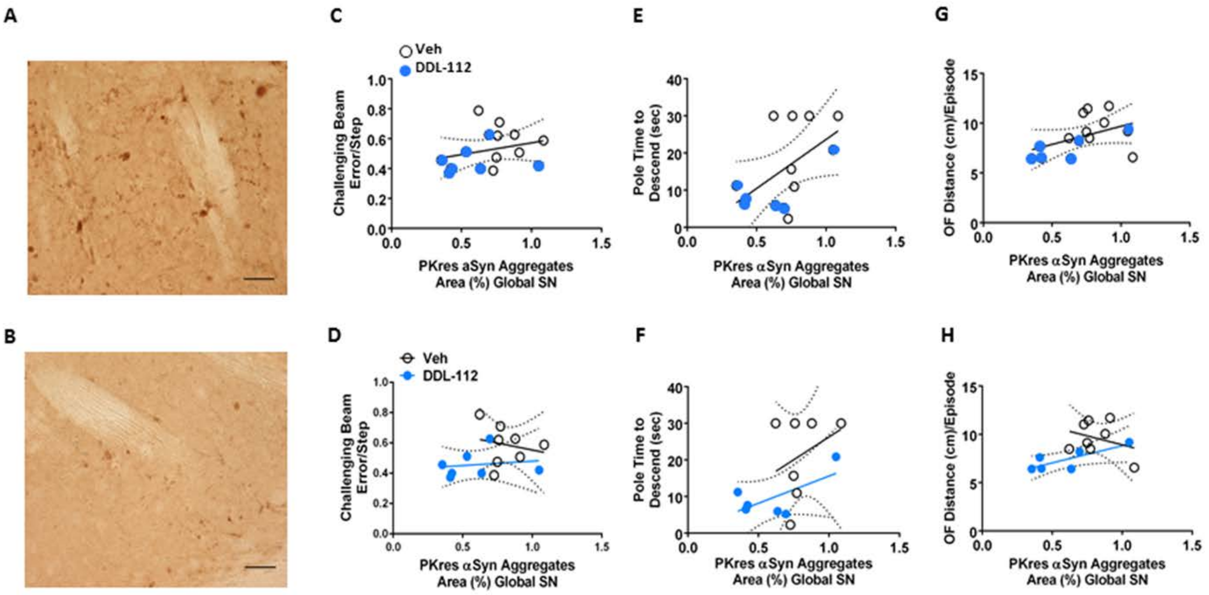

Fig. S3. *Chronic study PK-resistant αSyn in the substantia nigra (SN), and correlations between SN PK-resistant αSyn and behavior/motor function*. Representative images of PK-resistant αSyn in the dorsal medial region of the SN for (A) vehicle- and (B) DDL-112- treated Thy1-αSyn mice from the chronic study are shown (scale bar = 20 μM). (C, D) Linear regression analysis was performed for PK-resistant αSyn in the SN and the Errors/step in the challenging beam for all mice and by treatment, respectively. For all mice, in correlation analysis r = 0.2698, and for Veh r = -0.2008, and DDL-112 r = 0.1564. (E, F) Linear regression analysis is shown for PK-resistant αSyn in the SN and time to descend in the pole test. For all mice, in correlation analysis r = 0.5462, for Veh r = 0.3164, and for DDL-112 r = 0.6547. (G, H) Similar analysis is shown for PK-resistant αSyn in the SN and distance (cm) traveled per movement episode in OF; for all mice r = 0.4332, for Veh r = -0.2974, and for DDL-112 r = 0.8026.

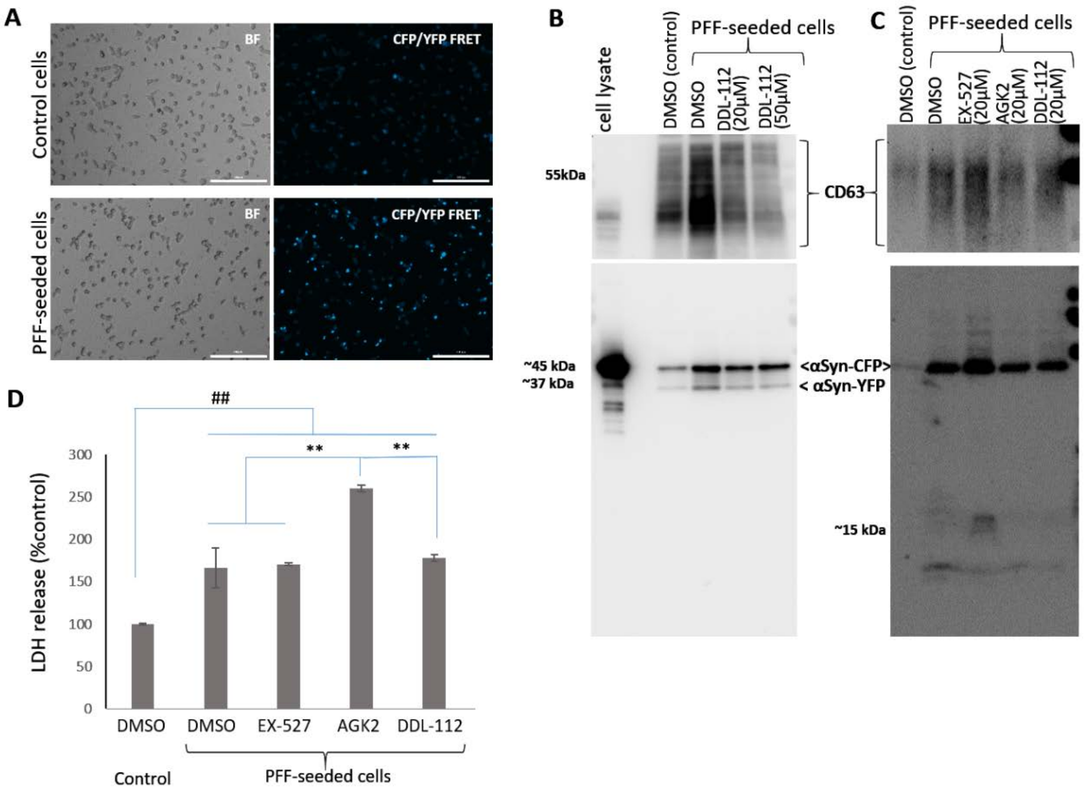

Fig. S4. *Evaluation of DDL-112, AGK2 (a SirT1 inhibitor), and EX-527 (a SirT2 inhibitor) effects on EV release and EV αSyn levels in vitro.* (A) Transfection of αSyn biosensor cells, expressing αSyn-CFP and αSyn-YFP constructs, with aSyn pre-formed fibrils (αSyn-PFF) induced intracellular aggregation of αSyn inside the cells and as a result CFP/YFP FRET signal by fluorescent microscopy. (B) IB analysis of CD63 and αSyn in EVs isolated from culture medium collected from PFF seeded αSyn biosensor cells treated with DDL-112 revealed dose-dependent decrease in EV/exosome release and exosome level of αSyn. (C) IB analysis of CD63 and αSyn in EVs isolated from control cultures and PFF seeded αSyn biosensor cell cultures treated with 20 µM of DDL-112, AGK2, EX-527, or vehicle (DMSO) for 48 hrs. (D) Lactate dehydrogenase (LDH) levels in culture medium collected from cells treated with the inhibitors are shown. Treatment with SirT1 inhibitor AGK-2 was more toxic to PFF-seeded cells compared to treatment with DMSO or other compounds.
